# Griffithsin: An Antiviral Lectin with Outstanding Therapeutic Potential

**DOI:** 10.3390/v8100296

**Published:** 2016-10-24

**Authors:** Sabrina Lusvarghi, Carole A. Bewley

**Affiliations:** Laboratory of Bioorganic Chemistry, National Institute of Diabetes and Digestive and Kidney Diseases, National Institutes of Health, Bethesda, MD 20892, USA; sabrinal@mail.nih.gov

**Keywords:** carbohydrate binding agent, viral envelope glycoproteins, multivalency, resistance, immunogenicity, HIV, HSV, HCV

## Abstract

Griffithsin (GRFT), an algae-derived lectin, is one of the most potent viral entry inhibitors discovered to date. It is currently being developed as a microbicide with broad-spectrum activity against several enveloped viruses. GRFT can inhibit human immunodeficiency virus (HIV) infection at picomolar concentrations, surpassing the ability of most anti-HIV agents. The potential to inhibit other viruses as well as parasites has also been demonstrated. Griffithsin’s antiviral activity stems from its ability to bind terminal mannoses present in high-mannose oligosaccharides and crosslink these glycans on the surface of the viral envelope glycoproteins. Here, we review structural and biochemical studies that established mode of action and facilitated construction of GRFT analogs, mechanisms that may lead to resistance, and in vitro and pre-clinical results that support the therapeutic potential of this lectin.

## 1. Introduction

A number of life-threatening human diseases are caused by viruses as diverse as human immunodeficiency virus (HIV), Ebola, yellow fever, Zika, influenza, and severe acute respiratory syndrome and Middle East respiratory syndrome corona viruses (SARS-CoV and MERS-CoV, respectively). Though these viruses are taxonomically and genetically distinct, they are all enveloped viruses and therefore possess a lipid bilayer that protects the viral capsid and genetic material that is inside the viral particle. Despite the efforts to eradicate these diseases, a number of them continue to affect us either as pandemic diseases or as outbreaks that appear from time to time. For example, according to the World Health Organization (WHO), by the end of 2015 there were nearly 37 million people infected with HIV, with two million new cases and about one million deaths occurring that year (http://www.who.int/). On the other hand, nearly 29,000 cases and over 11,000 deaths were reported during the most recent Ebola outbreak that started in 2014. SARS-CoV and MERS-CoV belong to the corona virus family that includes the common cold. Outbreaks of both viruses (SARS-CoV in Asia in 2003 and MERS-CoV in Saudi Arabia in 2012) led to several thousand cases of SARS and MERS, and hundreds of deaths. Efforts to create vaccines against these viral diseases have been successful in some cases such as yellow fever, but other cases like HIV have been more challenging.

Enveloped viruses have surface glycoproteins that mediate attachment and fusion with the target cell membrane [1,2]. These proteins constitute the first encounter with the host and the most exposed target that the immune system can attack; hence viruses have evolved to hide the features that would make them more susceptible to antibody neutralization. These strategies include hiding fundamental structural motifs through oligomerization or conformational occlusion, rapid mutation rates that lead to high sequence variability in non-essential regions such as variable loops, and extensive posttranslational glycosylation. Vaccine development against enveloped viruses has mostly focused on targeting the envelope glycoproteins primarily because they are the sole targets of neutralizing antibodies. Some viruses, including hepatitis C virus (HCV) and HIV, have thus far eluded vaccine efforts, mostly due to the high variability among strains, high mutation rates within a given virus, and physical masking of neutralizing epitopes [3,4]. Development of vaccines against diseases that appear as outbreaks, such as Ebola and SARS-CoV, are also challenging because of the lack of infected patient populations necessary for testing efficacy of vaccines and therapeutics. Other diseases such as yellow fever can be prevented by vaccination.

For those diseases in which vaccine development has been challenging, other therapies have nevertheless been developed. HIV is arguably the most studied viral disease; highly-active antiretroviral therapy (HAART) consisting of a combination of small molecule antiretroviral drugs has progressively improved the lives of patients in some parts of the world, and effectively extends life expectancy. Antiretroviral drugs target different proteins and enzymes essential for the virus life cycle. On the other hand, antibody therapeutics are emerging as powerful alternative forms of therapy against HIV and other diseases. Such therapies depend on isolation of neutralizing monoclonal antibodies, primarily from patient sera, that will bind specifically to the envelope glycoprotein of the virus. Another way to treat viral diseases that is currently being studied is through the use of proteins capable of targeting the glycans present on the surface of the envelope glycoproteins, namely, lectins [5,6]. Lectins are sugar-binding proteins that are ubiquitous, present in microorganisms, plants and animals. They participate in many important cellular processes including cell–cell interactions and protein folding. Some lectins provide protection to the host from other organisms. Lectins have been developed widely as probes to investigate cell surface structure and functions; they have also found applications as antiviral drugs and in the delivery of chemotherapeutic agents. A number of lectins capable of binding the high-mannose glycans commonly found in the surface of the envelope glycoproteins are currently under study to be used as microbicides [7,8]. Some of the most promising antiviral lectins include griffithsin (GRFT), cyanovirin (CV-N) and banana lectin (BanLec). Their use has been mostly suggested as antiviral microbicides, acting as a barrier to prevent transmission of HIV through their incorporation into vaginal and rectal gels, creams or suppositories. In such systems, they can bind the viruses and prevent viral entry and fusion to target cells, thereby preventing infection. This review summarizes the activity, biochemical, biophysical and structural data available for GRFT, one of the most potent anti-HIV agents reported to date.

## 2. Discovery and Recombinant Expression of Griffithsin

Griffithsin was discovered as an anti-HIV lead by researchers at the National Cancer Institute (NCI). GRFT was isolated from a marine red alga *Griffithsia* sp. present in the NCI Natural Products Repository. Mass spectroscopic and nuclear magnetic resonance (NMR) data indicated the active compound was a protein rather than a small molecule natural product. Its sequence was determined through a combination of N-terminal Edman degradation of the intact protein and N-terminal sequencing of peptide fragments obtained from endopeptidase and cyanogen bromide treatments [9]. The wild-type protein from the alga contained an uncommon amino acid of 151.05 Da at position 31 that was replaced by alanine (Ala) in recombinant protein preparations without affecting anti-HIV activity. GRFT has no homology to any other proteins previously reported. It has been shown to have anti-HIV activity against T cell tropic and macrophage-tropic viruses. It is capable of inhibiting cell–cell fusion between chronically infected and uninfected cells and its efficacy as an antiviral agent against other enveloped viruses has also been shown (see Section 6).

A large-scale expression system is essential for the development of GRFT as an affordable drug. To that end GRFT has been expressed recombinantly in different organisms (Table 1). It was first expressed recombinantly in *Escherichia coli* (*E. coli*) with a polyhistidine tag on the N-terminus to facilitate purification by metal affinity chromatography [9,10]. Expression of GRFT in *Nicotiana benthamiana* using an infectious tobacco mosaic virus (TMV)-based vector has yielded gram amounts of the protein [11,12]. Expression in rice seeds through the stable transformation of plants has also been reported [13]. Purification of GRFT has been also optimized, including the use of ceramic filtration followed by two-stage chromatography [11], and a combination of heat, magnesium chloride and bentonite, followed by a single chromatographic step [14]. Importantly, GRFT expression and purification has been proven robust, an essential feature in making a drug available for clinical testing.

## 3. Three-Dimensional Structure

**Figure 1:**
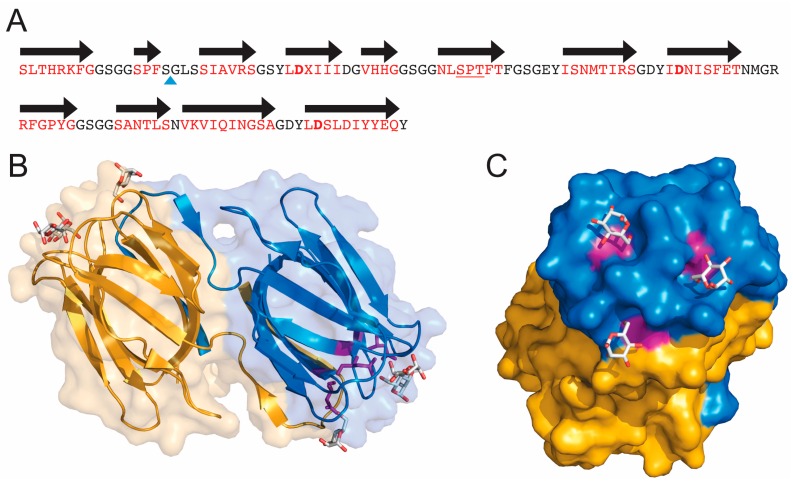
Sequence and three-dimensional structure of griffithsin (GRFT). (**A**) Sequence of wild-type GRFT. Amino acids located within beta strands are colored red and the black arrows correspond to secondary structure. X represents an unknown amino acid of mass 151.05 present in the natural alga-derived material. The blue triangle shows the site of the Gly-Ser insertion used to produce the monomeric version of GRFT (mGRFT). The three Asp residues located in the three mannose-binding sites are shown in bold; (**B**) Ribbon drawing showing the three-dimensional structure of GRFT. Each monomer of the domain swapped dimer is colored yellow or blue. Mannose residues bound to the carbohydrate binding sites are shown in stick representation. Aspartic acids present in the binding sites are colored purple; (**C**) Rotated view of GRFT showing the carbohydrate binding face and mannose binding sites. (**B**,**C**) were generated using the program Pymol (Delano Scientific LLC, Palo Alto, CA, USA) and protein data bank accession number 2GUD.

Griffithsin exists as a stable homodimer where each subunit contains 121 amino acids (Figure 1A). It has no cysteines in its sequence and no homology to any other proteins. Structures of GRFT in the absence of any ligand as well as in the presence of different monosaccharides and disaccharides have been solved by X-ray crystallography [15,16,17]. GRFT folds into a domain-swapped dimer (Figure 1B), where each subunit presents nearly perfect internal three-fold symmetry. The structure is composed by three repeats of an antiparallel four-stranded β-sheet [15] that superficially resembles a β-prism-I motif found in other lectins of the jacalin family. Two out of 12 β-strands (16 amino acids) swap from one monomer to the other to form a β-prism of three four-stranded sheets. Each subunit of the homodimer griffithsin is capable of binding three monosaccharides. Each binding site is located in an equilateral triangle, with each site separated by approximately 15 Å (Figure 1C). Crystal structures with the monosaccharides mannose, glucose and *N*-acetylglycosamine, and the disaccharides 1-6α-mannobiose and maltose have been reported [15,16,17]. When interacting with oligosaccharides, it has been shown that GRFT preferentially interacts with the terminal sugar [16]. Crystals with mannose have shown that all binding sites are almost identical and each one contains an aspartic acid (Asp) residue that makes extensive contacts with the sugar. These include Asp30, Asp70 and Asp112 that make hydrogen bonding interactions with O5 and O6 of mannose [15] (Figure 1C). Mutations of these residues to Ala do not affect the folding of the protein but weaken the binding to a mannose column [18].

**Figure 2:**
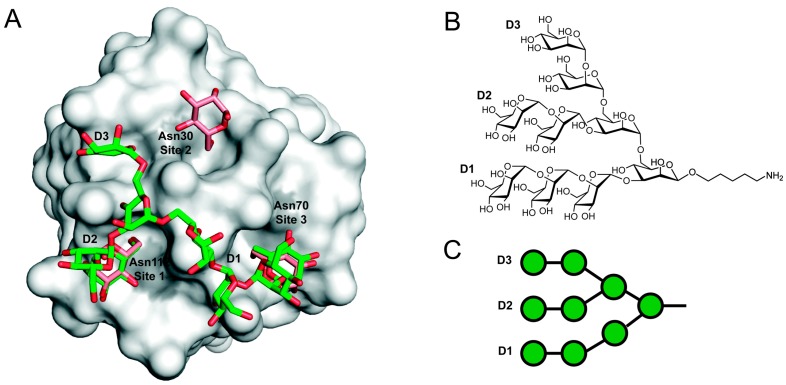
Model of a GRFT-Man9 complex. (**A**) Representation of the structure of monomeric GRFT (gray surface) bound to nonamannoside (green sticks), Protein Data Bank accession 3LL2. Aspartic acid residues from each of the three glycan-binding sites are highlighted in grey. The structures of a GRFT:synthetic nonamannoside complex and a 1:3 mGRFT:mannose complex have been superimposed to compare the mannose binding sites. Only the D1 and D2 terminal mannoses are bound to a single GRFT monomer. Figure was rendered using PyMol. (**B**) Chemical structure of nonamannoside showing the D1, D2 and D3 arms; (**C**) Symbol representation of nonamannoside.

Attempts to crystalize GRFT with oligosaccharides have been mostly unsuccessful because of extensive precipitation of the protein in the presence of the oligosaccharides [16]. This problem was solved by Moulaei et al. who were able to create a monomeric version of GRFT (mGRFT) by insertion of a short linker, Gly-Ser, that separates the β-strands that undergo intermolecular domain swapping [19]. A second mutation, leucine in position 2 to serine, was introduced to minimize the hydrophobic patch opposite to the swap region. A complex of mGRFT bound to a synthetic nonamannoside (Man9) that contains the nine mannose residues present in full-length mannonanose-di-(*N*-acetyl-D-glucosamine) (Man9GlcNAc2) but lacks the core. In that structure, they found two of the three arms of nonamannoside bound to two of the three mannose-binding sites (Figure 2). In particular, the terminal mannose units on the D2 and D1 arms were bound to sites 1 and 3 (as defined in ref [15]), respectively [19]. The third mannose-binding site, site 2, is occupied by the D2 arm of another nonamannoside in what likely is a more transient interaction [19]. This interaction was inconsistent with the model proposed by Ziókowska et al. [16] where it was proposed that all three arms of Man_9_GlcNAc_2_ could interact with GRFT with a one-to-one stoichiometry. In addition to the crystal structures, NMR assignments for the backbone atoms of GRFT in the absence of ligand and in the presence of mannose have been reported [18].

The crystal structures of GRFT:mannoside complexes suggested the importance of Asp residues present in each of the three mannose binding sites. Single mutations of any one of the Asp residues to Ala slightly weaken the affinity of binding to HIV-1 envelope glycoprotein gp120. However, the single mutations significantly decrease the potency in inhibition in single round HIV infection and cytopathicity assays as well as cell-to-cell transmission assays (cocultivation assays) [18,20]. To further study the importance of having three binding sites on each monomer, Xue et al. [21] designed a series of mutants where two monomers connected by an amino acid linker were expressed to form an obligate dimer. They compared a construct having all six binding sites (three from each monomer), with a mutant where three of the binding sites in one of the subunits were mutated to abolish binding to mannose (one-arm obligate dimer) and another mutant in which all six mannose binding sites were mutated. Binding studies showed that the obligate dimer as well as the construct containing mannose binding sites on only one subunit bound to gp120 with comparable affinities, whereas the mutant lacking all mannose binding sites did not bind gp120. In contrast, neutralization assays showed that the anti-HIV activity of the one-arm obligate dimer was reduced by several orders of magnitude compared to the wild-type obligate dimer [21].

Isothermal titration calorimetry (ITC) showed that binding to mono- and disaccharides is exothermic and entropically favored with micromolar *K*_D_ values. Enthalpies among different saccharides indicated favorable electrostatic contacts. Large changes in entropy were reasoned by large displacement of water molecules from the binding sites [16,17,19]. Interestingly, nonamannoside binding was largely enthalpically driven and entropically unfavorable, indicating the formation of more favorable contacts between the sugar and the protein. The unfavorable entropy of binding indicated that the multivalent interactions between GRFT and nonamannoside diminished the degrees of freedom of glycan and protein [19].

## 4. GRFT Fragments, Conjugates and Stability

### 4.1. Peptides Derived from GRFT

Using the structure and carbohydrate binding arrangement of GRFT, Micewicz et al. constructed a peptide, grifonin-1 (GRFN-1), that contained three covalently linked beta sheets and displayed three-fold symmetry [22]. Grifonin-1 inhibited laboratory strains of HIV and bound to viral glycoproteins. Though antiviral potency was reduced by many hundred-fold compared to GRFT, grifonin-1 nevertheless exhibited submicromolar half maximal inhibitory concentrations (IC_50_s) in single-round HIV infectivity assays.

### 4.2. GRFT-C37

To enhance the ability of GRFT to inhibit HIV infection, Kagiampkis et al. created a covalently linked fusion protein composed of GRFT and the known HIV entry inhibitor C37 [23]. C37 is a 37-amino acid peptide containing sequences from the C-terminal region of the HIV transmembrane protein gp41. C37 blocks HIV fusion by binding to the N-terminal helices of gp41 and preventing the formation of the six-helix bundle that leads to membrane fusion. The fusion constructs demonstrated 5–8-fold improvements in potency compared to GRFT alone in C-C chemokine receptor type 5 (CCR5)-tropic or C-X-C chemokine receptor type 5 (CXCR5)-tropic cell-cell fusion assays and single-round infection assays [23].

### 4.3. Tandemers

Following on their work with monomeric GRFT, Moulaei and co-workers created several GRFT tandemers [24]. The tandemer proteins were constructed by linking GRFT monomers to one another through covalent linkages formed by insertion of Gly-Thr-Gly linkers. The tandemers were analyzed by electron microscopy that showed the double and triple repeats to exist in a linear arrangement while the tandemer containing four mGRFT repeats formed a more globular structure. In HIV neutralization assays, improvements in potency were observed for the double and triple tandemers but potency plateaued there, with no increase in potency observed for the 4× mGRFT construct.

### 4.4. GRFT Stability

GRFT is an unusually stable protein, withstanding extraction procedures used in natural products’ isolation and purification. These may include exposure to organic solvents and repeated lyophilization. In addition to this physicochemical stability, its resistance to protease degradation was established in a study examining the stability of antimicrobial peptides. There, the authors showed that GRFT was resistant to digestion by eight out of nine different proteases and was degraded only by the metalloprotease elastase [25]. In addition, GRFT was not affected under bacterial degradation conditions.

## 5. Carbohydrate-Mediated Crosslinking

The importance of intermolecular crosslinking by GRFT has been suggested in several publications [18,19,20,21,26]. Evidence has been obtained from the protein perspective as well as from the glycan perspective. It has been shown that monomeric GRFT binds with comparable affinity to gp120, yet the IC_50_ for neutralization is several orders of magnitude weaker than wild-type GRFT [19]. Crystal structures with a synthetic nonamannoside have been obtained only for the monomeric GRFT construct. The mode of binding seen in the crystals was in disagreement with a model proposing all three terminal mannose units of Man_9_ interacting with each of the mannose-binding sites in GRFT [16,19]. The inability of Man_9_ to engage all three binding sites on GRFT was further supported by studies that showed that glycopeptides that mimic Man_9_ required distances greater than those separating the arms of natural Man_9_GlcNAc_2_ in order to engage all three binding sites simultaneously [26]. Most recently, cryo-electron microscopy and surface plasmon resonance studies of viral particles treated with GRFT or GRFT mutants demonstrated that the unique combination of multivalency, strict recognition of Man_9_ and homodimeric structure of GRFT led to bridging of viral particles and aggregation [27].

The ability of monomeric GRFT tandemers, linked together by Gly-Thr-Gly amino acid linkers, showed that two individual monomeric units were needed to recover (and improve by up to 5-fold) the potency of wild-type GRFT [24]. Three units of mGRFT improved the neutralization potential even further (5-fold more). However, no further improvements in potency were observed for tandemers containing a greater number of mGRFT domains [24]. Interestingly, dynamic light scattering showed that wild-type GRFT causes virus aggregation, however, monomeric GRFT as well as the mGRFT tandemers did not cause virus aggregation; in addition, cryo-electron microscopy showed that viruses exposed to wild-type GRFT had protein aggregates on their surface but immunostaining was not performed [24].

## 6. Antiviral Activity

**Figure 3:**
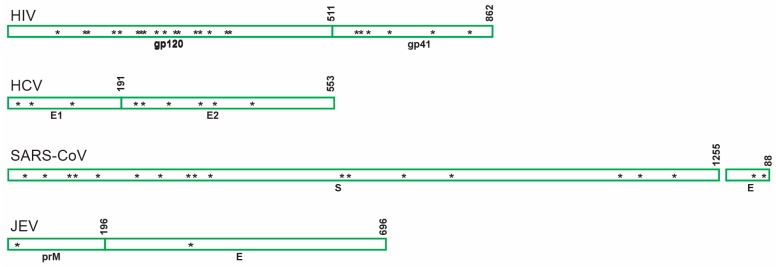
Schematic showing distribution of the predicted asparagine-linked glycosylation sites of envelope glycoproteins belonging to different viruses inhibited by GRFT. HIV: human immunodeficiency virus; HCV: hepatitis C virus; SARS-CoV: severe acute respiratory syndrome coronavirus; JEV: Japanese encephalitis virus; gp120: envelope glycoprotein gp120; gp41: transmembrane protein gp41; E1 and E2: envelope glycoprotein 1 and 2 respectively; S: spyke protein; E: envelope glycoprotein; prM: premembrane gycoprotein.

GRFT has been shown to have antiviral activity against a number of enveloped viruses as described in this section. Though the predicted number of glycans varies considerably among the viruses inhibited by GRFT (Figure 3), antiviral activity has nonetheless been attributed to carbohydrate-mediated binding to the respective viral envelope glycoproteins. A list of effective concentrations to inhibit 50% of virus infection (EC_50_) is presented in Table 2, with HIV being the most studied target.

### 6.1. HIV

HIV can infect cluster of differentiation 4 positive (CD4)^+^ target cells that include T cells, macrophages and dendritic cells (DC). HIV infection can occur cell-free via infection by HIV particles or by cell–cell contact between HIV-1 infected T cells or dendritic cells and non-infected CD4^+^ target T cells at a virological synapse [35]. These synapses are formed when high amounts gp120 present on the surface of HIV-infected cells bind the CD4 receptors on uninfected CD4^+^ cells. This interaction triggers the same conformational changes needed for further co-receptor binding, followed by gp41 mediated membrane fusion.

It has been shown that GRFT is capable of inhibiting in vitro infection of clade A, B and C viruses, with clade A being the least sensitive to GRFT [11]. Additionally, it was shown that the sensitivity to inhibition was highly dependent on the number of glycans on the surface of a gp120 [11]. It has been shown that single-cycle pseudotypes (TZM-bl cell neutralization assay) are generally more sensitive than neutralization in PMBC assays [36]. GRFT has been shown to inhibit the fusion between HIV-1 infected T cells and non-infected CD4^+^ T cells [9,37]. GRFT is also capable of preventing infection on cervical explants and cell-to-cell transmission on migratory cells from such explants [11]. Thus, GRFT has the ability to block HIV infection in numerous assays and platforms.

Interestingly, even though the monomeric GRFT had comparable affinity towards nonamannoside, it showed decreased ability to bind to gp120 and even more dramatic decrease in inhibiting HIV infection in CEM-SS cells [19]. This indicates that crosslinking of multiple high-mannose sugars is responsible for the high potency of the lectin GRFT.

HIV transmission is believed to be mediated predominantly by transmitted/founder (T/F) viruses. These viruses show differential glycosylation when compared with chronic viruses. The effect of GRFT on T/F viruses has been measured and it has been found that there is a great variability on the IC_50_ values for GRFT against T/F viruses [38]. Interestingly, even though there was no correlation between the number of possible *N*-linked glycosylation sites and the sensitivity to GRFT for T/F viruses, there seemed to be a correlation between the number and location of high-mannose glycan sites and the resistance to GRFT [38]. Lastly, it has been shown that GRFT can block HIV-2 replication in MT-4 cell cultures with EC_50_ values in the sub-nanomolar range [29].

#### 6.1.1. Mode of Action

##### Binding to HIV-1 Envelope Glycoprotein gp120

GRFT has been shown to bind the HIV envelope glycoproteins thereby blocking CD4 binding as well as binding of other anti-HIV antibodies. These include the conformation-specific monoclonal antibody (mAb) 48d that binds to the CD4-induced epitope on gp120, and the carbohydrate-specific mAb 2G12 [9,36]. Interestingly, binding of the lectin cyanovirin-N (CV-N) to gp120 was shown to inhibit subsequent binding of GRFT; in contrast, GRFT binding to gp120 does not block subsequent binding by CV-N [9]. This could suggest that GRFT has a smaller recognition epitope, or lower stoichiometry of binding compared to CV-N. However, ITC experiments showed that a total of approximately 10 GRFT units are capable of binding to a single gp120 glycoprotein [30]. In regard to timing of inhibition, GRFT has been shown to initiate antiviral activity immediately upon contact with HIV-1. This represents an advantage when compared with other antiretroviral agents, such as the nucleoside phosphonate (*R*)-9-(2-phosphonylmethoxypropyl)adenine (PMPA) that must be administered as a prodrug ester [28].

##### Effects of GRFT on CD4 and Co-Receptor-Binding Sites

It has been shown that GRFT competes with the glycan-specific antibody 2G12 [36], a mAb that binds the high mannose patch on gp120, but enhances binding of antibodies specific for the CD4 binding site (CD4bs) including mAbs b12 and b6 and the chimeric molecule CD4-IgG2 [39]. mAb b12 is a broadly neutralizing antibody whereas mAb b6 is non-neutralizing. CD4-IgG2 is fusion protein used as a surrogate of CD4 where the variable region of an IgG2 molecule has been replaced with the soluble region of the CD4 receptor. Deletion of the glycan in position 386 decreases the GRFT-mediated increase in b12 binding [39]. This data was supported by the observation that GRFT and mAb b12 act synergistically and that the deletion of the glycan at asparagine (Asn) 386 prevents the synergistic effect [39].

In contrast with the enhancement of binding to the CD4 binding site, it was shown that GRFT inhibits binding to the co-receptor binding site-specific mAb 17b without directly occluding the binding site [39]. Alexandre et al. show that the binding of GRFT to the glycans on gp120 likely interferes with the CD4-induced conformational changes needed for co-receptor binding [39]. Another study showed that the presence of GRFT partially inhibits the binding of CD4-IgG2, the CD4-binding site mAbs b12 and VRC01 and the CD4-induced epitope mAb 17b in the presence or absence of soluble CD4 (sCD4), suggesting that the presence of GRFT might compromise the exposure of the CD4 binding site [40].

To further investigate the effect of GRFT on exposing the CD4bs, Xue et al. showed that all six mannose-binding sites were needed to expose this site, as mutants lacking mannose-binding sites on one domain did not show CD4bs exposure [21]. They further showed that binding of GRFT to gp120 induces shedding, and this effect is more marked for clade B strains than clade C [21].

##### Inhibition of DC-SIGN Binding

Dendritic cells are antigen-presenting cells responsible for capture and degradation of pathogens that enter the mucosal environment. DCs also play an important role in viral transmission. DC cells present a DC-SIGN (dendritic cell intercellular adhesion molecule 3 (ICAM-3) grabbing non-integrin) receptor on their surface. DCs migrate to the lymph nodes where the DC-SIGN receptor mediates the transmission to naïve uninfected CD4^+^ T cells. It is believed that the interaction of HIV-1 with dendritic cells through the DC-SIGN receptor mediates the transfer of HIV-1 to the lymph nodes where it infects CD4^+^ T cells. The DC-SIGN mediated transfer of HIV to target cells involves binding of the virus to the DC-SIGN receptor, a process that occurs through interactions between high-mannose glycans on the surface of the envelope glycoprotein with DC-SIGN, followed by transfer to CD4^+^ target cells for infection. GRFT has been shown to inhibit both processes [37,41]. In addition, single-site mutations on the mannose-binding site of GRFT have been shown to significantly diminish DC-SIGN associated cell-to-cell transmission [20]. Competition assays have shown that GRFT inhibits the binding of DC-SIGN to gp120 [20,40]. GRFT is not cytotoxic to DCs and does not trigger DC apoptosis or maturation [42].

#### 6.1.2. HIV Resistance

**Figure 4:**
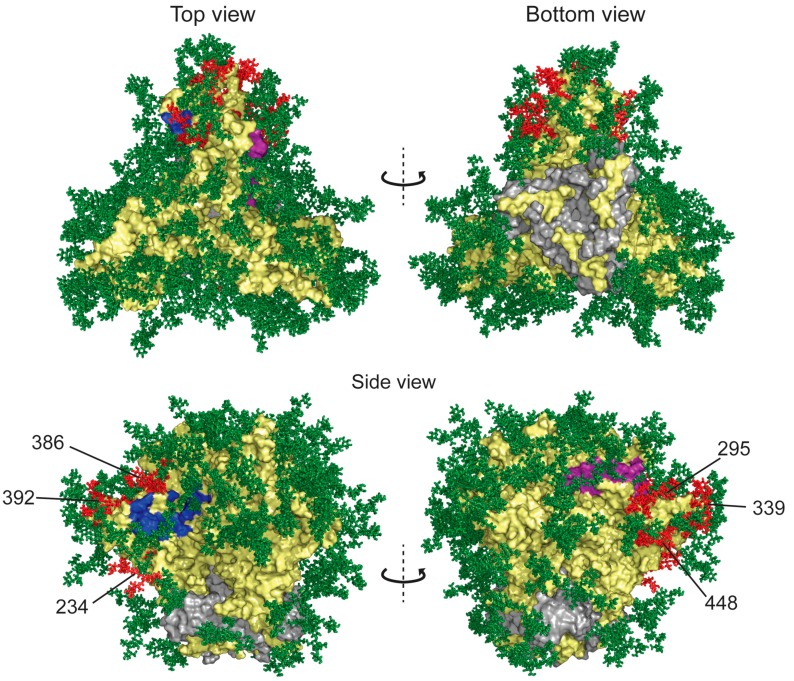
Structure of glycosylated HIV-1 gp120 trimer with full-length glycans modeled onto the protein. The surface envelope glycoprotein gp120 and transmembrane protein gp41 are shown as yellow and gray surfaces, respectively. CD4 receptor and co-receptor binding sites are colored blue and purple, respectively. Glycans found to be deleted in resistance studies are colored red (glycans 234, 295, 339, 386, 392 and 448). Other glycans shown to be deleted in GRFT-resistant strains but not present in this structure include glycans 230, 241 and 298. Model was rendered with Pymol. Model was constructed by C. Soto, PDB Accession Number 5FYK [44] and rendered with Pymol.

Several excellent studies investigating mechanisms of GRFT resistance in HIV have been published. Not surprisingly, one strategy used by HIV against all lectins as well as glycan-targeting mAbs involves deletion of glycosylation sites (Figure 4). Alexandre et al. performed an extensive analysis comparing the IC_50_ values of observed for GRFT against numerous HIV-1 subtype A and B strains, with the glycosylation of naturally-occurring viruses [36]. Their results showed that viruses missing glycosylation at both Asn 234 and 295 sites were ~20–100× less sensitive to GRFT. However, the absence of just one of these glycosylation sites did not affect the sensitivity. In addition, the number of missing glycans did not correlate with sensitivity to GRFT, but the location of glycosylation sites had a clear effect on efficacy. Huang et al. [43] on the other hand investigated the role of glycans at Asn 295 and 448 in the background of a subtype C HIV strain MWS2. Mutants with a single deletion in either of these two glycosylation sites showed decreased susceptibility to GRFT (20–100× less sensitive), and double mutants showed an even more pronounced decrease in GRFT sensitivity. In a different study, strains lacking the glycan in position 295 showed lower sensitivity to GRFT and strains missing glycans 234 and 295 significantly reduced sensitivity [21].

In a separate study, the Morris and Moore laboratories examined the mechanisms of resistance of an HIV-1 subtype C strain to lectins including GRFT. They found that viruses cultured in the presence of increasing concentrations of GRFT evolved deletions of several glycans including those in positions 339, 230, 234, 241, 298, 392 and 448. Insertion and deletion of amino acids near the glycosylation sites in the V4 loop of gp120 that would be expected to alter glycosylation were also observed [45]. Importantly, when natural resistance evolves, these studies and others suggest that multiple glycosylation site mutations are required for a virus to become resistant to GRFT, and the level of resistance is only 3–10 fold compared to the wild type [45]. In another study examining the effect of a different lectin, *Oscillatoria agardhii* agglutinin (OAA), GRFT-resistant HIV-1 IIIB strains were generated and resulted in deletions of glycans in positions 230, 234, 295 386 and 448, and these multiple mutations decreased GRFT susceptibility by more than 10,000 fold [46]. Though studies examining corresponding effects of these glycosylation site deletions on viral fitness are limited, some studies report decreased infectivity in recombinant strains.

#### 6.1.3. Synergy Studies

A number of laboratories have studied synergistic activities between GRFT and other proteins including antibodies and lectins as well as commonly used antivirals.

##### Antibodies

The monoclonal antibody 2G12 has been used in several studies because it recognizes a carbohydrate-dependent epitope comprising a cluster of high mannose glycans. These include glycans at Asn residues 332, 339 and 392, sites that are suggested to be targets of GRFT on the basis of resistance profiles. In competition enzyme-linked immunosorbent assays (ELISA), capture of GRFT-treated virus by immobilized 2G12 showed these proteins to be competitive with one another [36]. However, combinations of GRFT and 2G12 were shown to be synergistic against R5 HIV-1 strain BaL in PBMC cultures [29]. In other studies, GRFT synergized with the CD4 binding site mAbs b12 and VRC01, with plasma from HIV-positive individuals, and with the anti-CCR5 antibody mAb PRO140 [29,39,47]. GRFT was also found to be synergistic with the soluble version of the CD4 receptor, sCD4 [39].

##### Lectins

Owing to their potency and general potential as topical microbicides, numerous studies have looked at the effects of synergy between GRFT and other lectins, including mannan-binding lectin (MVN), banana lectin (BanLec), *hippeastrum* hybrid (amaryllis) lectin (HHA), and *galanthus nivalis* lectin (GNA) in virus infectivity assays [29]. Synergy is more pronounced for some lectin resistant HIV-1 strains [29]. BanLec has also shown to have additive effects with GRFT in cell-to-cell transmission assays [37]. Interestingly, GRFT showed additive effects with OAA whereas it showed antagonism with the lectin hybrid OAA-homologous protein (OPA) [46]. As with the synergy studies employing GRFT and HIV-neutralizing antibodies, the type of assay may play a role in the observed outcome.

##### Tenofovir, Maraviroc and Enfuvitride

It has been shown that GRFT synergizes with known HIV-1 drugs, including the nucleotide reverse transcriptase inhibitor tenofovir, the CCR5 HIV co-receptor antagonist maraviroc, the gp41 fusion inhibitor enfuvitride and the specific CXCR4 antagonist AMD3100 against using different HIV-1 clades in virus infectivity assays as well as cell–cell transmission assays [37,48]. These drugs individually, or combined with other anti-HIV drugs, have been shown as promising candidates for microbicide development. Combinations of GRFT and these molecules are believed to have great potential in the development of microbicides [48]. Tenofovir has been shown to have a synergistic effect with GRFT against HIV-2 [29].

### 6.2. Other Viruses

#### 6.2.1. Hepatitis C Virus (HCV)

GRFT has been shown to inhibit infection of HCV to hepatocytes in vitro as well as cell-to-cell transmission. This has important clinical relevance because, following liver transplantation in HCV-infected patients, reinfection of the new liver graft commonly occurs [31,32]. Co-immunoprecipitation studies showed that GRFT interacted with the envelope glycoprotein E1E2, and prevented subsequent interaction with one HCV receptor, CD81 [31]. In contrast, ELISA experiments showed that GRFT did not inhibit the binding of E2 to CD81 [32]. The infectivity is significantly inhibited by nonamannoside, indicating that like in the case of HIV, GRFT interacts with the high-mannose glycans on the surface of HCV [31]. GRFT treatment in chimeric mice with livers engrafted with functional primary human hepatocytes showed significantly lower virus titers when compared with treatment with a GRFT mutant where the binding sites have been mutated to prevent binding to glycans [31,32]. Even though mutations occurred upon exposure of HCV to GRFT, attempts to generate resistance against GRFT failed to detect specific envelope mutations that conferred resistance to the lectin [49].

#### 6.2.2. Severe Acute Respiratory Syndrome-Related Coronavirus (SARS-CoV)

The *Coronaviridae* are enveloped positive-strand viruses that are the causative agent of severe acute respiratory syndromes (SARS). Well-known strains and outbreaks include the SARS-CoV outbreak of 2002 and the MERS-CoV outbreak of 2012. GRFT has been shown to inhibit corona virus replication and cytopathicity induced by the SARS-CoV, as well as other *Coronaviridae* viruses [15,30]. In particular, GRFT inhibited different strains of SARS-CoV infection in Vero 76 cells with an EC_50_ in the low nanomolar range, with minimal toxicity on control cells [30]. GRFT binds glycans on the surface of the SARS-CoV spike glycoprotein S (S). A total of three molecules of GRFT are capable of binding the S in a dose dependent manner with very high affinity, a lower number when compared with HIV-gp120 probably due to the lower number of high-mannose glycans on the surface of S. Interestingly, such interaction does not inhibit the binding of the SARS-CoV S glycoprotein to the host cell human receptor angiotensin I converting enzyme 2 (ACE2) [30].

Studies on mouse-adapted MA15 SARS-CoV showed that mice that received daily doses of intranasally administered GRFT had 100% survival rate, with no loss in weight, improved lung histopathology scores, and reduction of lung tissue virus titers, compared to the control group that experienced weight loss and had only a 30% survival rate [30]. Further studies might prove GRFT as a good candidate molecule to treat respiratory infections [30].

#### 6.2.3. Japanese Encephalitis Virus (JEV)

Japanese encephalitis virus is a mosquito-borne flavivirus, similar to dengue, yellow fever and Zika. The envelope glycoprotein of JEV contains two potential glycosylation sites that are important for virus infection. It has been shown that GRFT can inhibit infection of baby hamster kidney (BHK)-21 cells with IC_50_ in the nanomolar range [33], and that such inhibition is due to the interaction between GRFT and the glycosylated viral proteins (Envelope, E and premembrane, prM) [50]. Additionally, mice challenged with lethal doses of JEV showed reduced virus titers in the mouse brain and 100% survival rate compared with a 0% survival rate in the control group [33].

#### 6.2.4. Herpes Simplex Virus Type-2 (HSV-2)

In in vitro studies, GRFT showed weak-to-moderate activity in blocking HSV-2 entry, yet it was capable of inhibiting cell-to-cell spread with IC_50_s in the nanomolar range [34,42]. In in vivo studies using a murine model, a gel containing 0.1% GRFT significantly protected mice from HSV-2 challenge compared to the control group. Further, it was shown to prevent viral spread post-challenge, and continued to protect when virus was introduced in seminal plasma. Though GRFT treatment led to a small decline in epithelial barrier integrity in polarized cell cultures, no increase in HIV migration was observed establishing its safety for HSV-2 prevention [42]. In vitro binding and immunoprecipitation studies suggest GRFT blocks HSV-2 infection by interacting with one of the four glycoproteins involved in HSV-2 entry, namely glycoprotein D [34].

#### 6.2.5. Human Papilloma Virus (HPV)

In vitro experiments have shown that GRFT has anti-HPV activity ranging from the high nanomolar to the low micromolar range [34]. HPV is a non-enveloped virus. Interestingly, it was found that GRFT does not block the initial attachment of the virus to the cell; instead, it acts in a late step in the HPV entry process [34]. Data suggests that GRFT interacts with the HPV secondary receptor α6 integrin, decreasing its availability on the cell surface. GRFT decreased HPV 16 pseudovirus (PsV) vaginal infection in a murine model [34].

#### 6.2.6. Gamma- and Delta- Retroviruses

In a study investigating the effects of three lectins on delta and gamma retroviruses including human T cell leukemia virus, GRFT had no effect [51].

## 7. Activity against Protists

Trichomoniasis is a sexually transmitted parasitic disease, caused by *Trichomonas vaginalis*. Development of microbicide agents against HIV can benefit also the prevention against *Trichomonas*. GRFT binds to the surface of *Trichomonas* and *Tritrichomonas* and causes flagellated trichomonads to undergo self-aggregation and precipitation [52]. Such interaction is likely due to the binding of GRFT to unprocessed glycans on the surface of the parasite. Topical application of GRFT during infection with *Tritichomonas* in mice reduced the recovery of parasites in mice, but it did not eliminate it [52].

## 8. Toxicity and Immunogenicity of GRFT

The cytotoxicity profile of GRFT has been studied extensively. While GRFT is by now well known to prevent infection by a number of diverse viruses, there has been no sign of cellular toxicity against a variety of cells even at concentrations as high as 500 nM [28]. If used as an antiviral therapeutic, the mode of GRFT administration may be determined by the mechanism through which the virus infects new hosts. For example, in the case of HIV, its most promising application is as a topical microbicide. Microbicides do not carry substantial risk of systemic side effects because they are unlikely to be absorbed efficiently. Alternatively, in respiratory infections via SARS-CoV, GRFT could be administered as an intranasal spray, or intravenously in the case of HCV.

Studies have shown that GRFT is stable and maintains similar anti-HIV activity after incubation for long periods of time in a variety of environments, including in the acidic (pH 4–6) cervical/vaginal lavage fluids from pig-tailed macaques [28]. GRFT binds the outermost layer of the squamous epithelium (human cervical epithelium) [53]. However, no loss in cell viability of endocervical and ectocervical cell lines was observed even at doses of 1 mg/mL GRFT. In addition, GRFT does not induce cell proliferation in cervicovaginal cell lines in contrast with other lectins that show high cytotoxicity and significant mitogenic activity [53]. Treatment of human cervical explants as well as cultured human endocervical, ectocervical and vaginal cell lines with GRFT, or intravaginal GRFT treatment of rabbits, have shown no significant perturbations on the levels of cytokines and chemokines [11,53]. RNA microarray analysis of cultured ectocervical cells line showed that GRFT treatment has minimal alteration in the gene expression profile [53]. An in vivo rabbit vaginal irritation (RVI) assay showed good safety profiles for GRFT, with no inflammatory responses or damage to epithelia. RVI testing is required by the Food and Drug Administration (FDA) to proceed for clinical testing [11].

HIV can infect PBMCs, which are often used in evaluating HIV infectivity. GRFT binds the surface of PMBCs and prevents viral replication even after washing the cells [53]. However, exposure of human PBMCs for up to three days showed no mitogenic activity, that is, it does not stimulate lymphocyte proliferation [11,53]. GRFT does not stimulate the expression of PMBC activation markers of immune activation (namely, markers for T cell activation) and it has minimal effect on cytokine and chemokine release on PMBC [53].

GRFT has also been proposed to treat respiratory infections such as SARS. In such cases, the protein would be administered intranasally. Preliminary studies where GRFT was administered intranasally against SARS-CoV infected mice showed that the levels of cytokines decreased compared those infected with SARS-CoV alone [30]. Perivascular infiltrates were observed on GRFT-treated mice, however, no further analysis was performed [30].

Subcutaneous injection has been tested to show the potential of GRFT to treat HCV. Subcutaneous injection of GRFT in immunodeficient transgenic mice indicated that the lectin remained bioavailable even after 18 days, with only mild transient alterations in health parameters such as body weight and morbidity scores. However, prolonged dosing had deleterious effects on the overall health of these mice [32]. The authors of this study suggest that this could be a consequence of the inherent fragility of the mouse model and not toxicity to implanted human hepatocytes [32]. Treatment of HCV-challenged transgenic mice showed a 2.5 log reduction in the HCV viral titer for the GRFT-treated mice [32]. In another HCV animal model, subcutaneous injections into chimeric mice harboring human hepatocytes in their livers resulted in two out of six animal deaths; similar to the previous study, the authors suggest that this is likely due to the fragile nature of the mice used in this model rather than lectin-induced toxicity [31]. Lastly, intraperitoneal administration of GRFT to mice before a lethal dose of JEV resulted in 100% survival whereas none of the mice in the control group survived [33].

Pharmacokinetic properties of GRFT were further tested in two rodent models. Subcutaneous injections of high amounts of GRFT (50 mg/kg) showed that GRFT persists in plasma for at least two weeks, and sera tested in HIV infectivity assays demonstrated that the GRFT in sera retains antiviral activity during this time. All animals survived the high dose GRFT treatment, and some accumulation in spleen, kidney and liver was observed [54]. Although minimal signs of toxicity were seen, juvenile animals showed weight loss compared to control animals. Importantly, the authors were unable to detect anti-GRFT antibodies in sera even in the high dose animals [54].

Another study assessed the immunogenicity of HIV-1 gp120 and Gag, in the presence and absence of GRFT [40,54]. Immunization of mice with a gp120-GRFT complex enhanced the anti-gp120 response compared to mice immunized with gp120 alone, but immunization with a non-glycosylated Gag-GRFT complex had no effect on the levels of anti-Gag antibodies compared to immunization with Gag alone. Interestingly, an increased anti-GRFT immune response was observed in mice immunized with gp120-GRFT but not the Gag-GRFT complex, leading the authors to conclude that immunization with GRFT-bound gp120 may improve the humoral immune response to gp120 [40].

## 9. Conclusions

GRFT belongs to a group of lectins capable of inhibiting HIV infection as well as infection by other enveloped viruses. GRFT binds the terminal mannose units present on high mannose oligosaccharides present on the surface of various viral envelope glycoproteins. Structural studies by X-ray crystallography and NMR have shown that the unique structure and distribution of mannose binding sites engender GRFT with an unprecedented ability to block HIV at much lower concentrations than other anti-HIV drugs. Even though GRFT has three mannose binding sites on each subunit, it has been shown that only two of them can be simultaneously engaged by the Man_9_ oligosaccharide, leaving the third binding site available for binding a second Man_9_ molecule. This precise distribution of carbohydrate binding sites together with the multivalent character of GRFT arising from its homo dimeric structure leads to the formation of a complex crosslinked network, likely one of the determining factors for its picomolar activity.

Given that the primary binding site of GRFT comprises the carbohydrates present on the surface of HIV-1 gp120, it was reasonable to evaluate the activity against other enveloped viruses. All the viruses that have been shown to be sensitive to GRFT inhibition contain moderate- to heavily-glycosylated envelopes, with a high density of high-mannose oligosaccharides present on many. The therapeutic potential of GRFT against these viruses will depend on the mode of entry of each virus. In closing, a growing body of literature has now established the remarkable potency, broad-spectrum anti-viral activity and safety profile of GRFT supporting its development as a microbicide, especially for diseases where preventative measures are unavailable.

## Figures and Tables

**Table 1:** Recombinant expression of griffithsin.

Expression System	Yield	Recovery after Purification	Ref.
*E. coli* BL21(DE3)—shake flasks	12 mg/L		[10]
*E. coli* BL21(DE3)—fermenter	819 mg/L	542 mg/L	[10]
Tobacco leaves (*Nicotiana benthamiana*)	1 g/kg leaf	300 mg/kg	[11]
Rice seeds (*Oryza sativa* endosperm)	301 mg/kg dry seed	223 mg/kg	[13]

**Table 2:** Effective concentrations to inhibit 50% of virus infection (EC_50_) of GRFT against different viruses.

Sample	Virus ^a^	Target Cell ^b^	EC_50_ (nM)	Ref.
Native GRFT	HIV-1_RF_	CEM-SS	0.043	[9]
Recombinant GRFT	HIV-1_RF_	CEM-SS	0.039	[9]
Native GRFT	HIV-1_RoJo_	PBMC	0.63	[9]
Native GRFT	HIV-1_ADA_	Macrophage	0.5	[9]
Native GRFT	HIV-1_Ba-L_	Macrophage	0.098	[9]
Recombinant GRFT	HIV-1_Ba-L_	PMBC	0.01	[28]
Recombinant GRFT	HIV-1_LAI_	MT-4	0.01	[28]
Recombinant GRFT	HIV-2_ROD_	MT-4	0.17	[29]
Recombinant GRFT	SIV _mac251_	CEMx174	0.35	[28]
Recombinant GRFT	SARS-CoV_200300592_	Vero 76	280	[15]
Recombinant GRFT	SARS-CoV_200300592_	Vero 76	960	[15]
Recombinant GRFT	SARS-CoV_Urbani_	Vero 76	48	[30]
Recombinant GRFT	SARS-CoV_Tor-II_	Vero 76	48	[30]
Recombinant GRFT	SARS-CoV_CuHK_	Vero 76	61	[30]
Recombinant GRFT	SARS-CoV_Frank_	Vero 76	94	[30]
Recombinant GRFT	HCV_JFH1_	Huh-7	13.9	[31]
Recombinant GRFT	HCV_JFH-1_	Huh 7.5.1	0.4	[32]
Recombinant GRFT	JEV	BHK-21	20	[33]
Recombinant GRFT	HPV_16 PsV_	HeLa	1390	[34]
Recombinant GRFT	HPV_18 PsV_	HeLa	428	[34]
Recombinant GRFT	HPV_45 PsV_	HeLa	928	[34]

^a^ HIV-1: human immunodeficiency virus type 1; HIV-2: human immunodeficiency virus type 2; SIV: simian immunodeficiency virus; SARS-CoV: severe acute respiratory syndrome corona virus; HCV: hepatitis C virus; JEV: Japanese encephalitis virus; HPV: humanpapillomavirus. ^b^ CEM-SS: human T-lymphoblastoid cell line; PBMC: peripheral blood mononuclear cell; BHK-21: baby hamster kidney fibroblasts; MT-4 human T cells isolated from a patient with adult T cell leukemia; CEMx174: somatic cell hybrid culture between CEM and B cell like 174 both of human origin; Vero 76: kidney epithelial cells extracted from an African green monkey; Huh: differentiated hepatocyte-derived carcinoma cell line.
